# Healthcare professionals’ views on the accessibility and acceptability of perinatal mental health services for South Asian and Black women: a qualitative study

**DOI:** 10.1186/s12916-023-02978-5

**Published:** 2023-10-02

**Authors:** Kiren Bains, Sarah Bicknell, Nikolina Jovanović, Maev Conneely, Rosemarie McCabe, Alex Copello, Jessica Fletcher-Rogers, Stefan Priebe, Jelena Janković

**Affiliations:** 1 Birmingham and Solihull Mental Health NHS Foundation Trust, Birmingham, UK; 2 Centre for Psychiatry and Mental Health, Wolfson Institute of Population Health, Queen Mary, University of London, London, UK; 3 East London NHS Foundation Trust, London, UK; 4 Unit for Social and Community Psychiatry (WHO Collaborating Centre for Mental Health Service Development), Queen Mary University of London, London, UK; 5 School of Health and Psychological Sciences, City University of London, London, UK; 6 School of Psychology, University of Birmingham, Birmingham, UK

**Keywords:** Perinatal mental health, Maternity care, Healthcare professionals, Ethnicity, Medical staff, Nursing staff, Midwifery, Qualitative research, Mental health, Health inequalities

## Abstract

**Background:**

Perinatal mental illness affects one third of new and expectant mothers. Individuals from ethnic minority groups experience higher rates of mental health problems and higher suicide rates. Despite this, women from ethnic minorities—Black and South Asian women in particular—are less likely to receive support from mental health services in the perinatal period. Healthcare professionals (HCPs) who have contact with women during this period have a unique perspective, and their views may provide insights to understand and remedy this health inequality. This study aimed to identify healthcare professionals’ views on the current accessibility and acceptability of perinatal mental health services, and ways of improving services by addressing the barriers for these women.

**Methods:**

Semi-structured interviews were conducted with twenty-four healthcare professionals who work with patients in the perinatal period. Purposive sampling was used to select HCPs from a range of different professions (including mental health staff, midwifery, primary care, social care). The data were analysed using Framework Analysis.

**Results:**

Three main themes were identified from the data: (1) lack of awareness and understanding of perinatal mental illness and service structure in both healthcare professionals and patients; (2) patients’ relationships with family, friends and healthcare professionals can both hinder and facilitate access to services; (3) healthcare professionals encourage raising awareness, flexibility, developing shared understandings and questioning assumptions to improve the accessibility and acceptability of services.

**Conclusion:**

Key insights into explaining and remedying the health inequalities observed between ethnic groups were proposed by healthcare professionals. Recommendations included sharing information; taking steps to ensure each woman was considered as an individual in her relationship with her culture, ethnicity and childrearing practices; and healthcare professionals addressing their possible unconscious biases through engaging in personal reflexive practices. Reasons these are currently not being implemented deserve further research, and the potential of novel roles such as peer support workers in bridging the space between ideals and practice needs further investigation.

**Supplementary Information:**

The online version contains supplementary material available at 10.1186/s12916-023-02978-5.

## Background

Policy in the UK stipulates that women should be asked about their mental health and wellbeing in pregnancy and after giving birth to detect possible mental health difficulties to prevent serious harmful outcomes [1]. However, estimates continue to suggest that mental health problem identification in pregnancy may be less than 50% [1, 2]. Research suggests that women who belong to an ethnic minority group in the UK are less likely to be asked about their mental health and offered treatment and support [3, 4]. There is a large gap between policy and practice which is having a harmful impact on women, and a more pronounced impact on women from ethnic minorities.

A recent study conducted as part of the current project identified Black and South Asian women as being particularly vulnerable: more likely to be admitted against their will, and less likely to access routine mental health services in the perinatal period (during pregnancy and first postnatal year) [4]. The reasons for this are not well understood. Research observing and interviewing health visitors in the postnatal period found that assessment of depression in the postnatal period is inconsistent and that women from minority ethnic groups were less likely to be assessed for postnatal depression [5].

A recent systematic review looking at women’s access to mental health services during the perinatal period identified barriers at four levels: individual, organisational, sociocultural and structural [6]. These multilevel complex barriers need to be addressed at different stages of the pathway to improve access to mental healthcare [6]. To be able to tackle the current health inequalities, the solution will need to target these different levels and be informed from insights from various perspectives. Healthcare professionals could provide insight at all levels to determine the challenges faced by ethnic minority women.

In the UK, specialist perinatal mental health services (PMHS) are designed to focus on the prevention, detection, early intervention and treatment of mental health problems during pregnancy and the postnatal period. Women obtain support from perinatal services through a variety of different pathways, from general practice, secondary mental healthcare and through midwives [1]. The healthcare professionals (HCPs) working across these pathways to accessing care have a unique, ‘bird’s eye’ view of the complex processes and mechanisms at play that are resulting in current health inequalities across ethnic groups. The healthcare professionals, from GPs to mental health midwives, will each see different parts of women’s pathway to care and will for this reason have different insights. Examining each facet, through different professionals’ perspectives, is important to be able to uncover and understand, with a view to ultimately redress, the current inequalities in access to support experienced by Black and South Asian women. Existing literature suggests barriers exist for women from ethnic minority groups to access mental healthcare support. Examples of factors are the influence of culture, experiences of services and awareness and beliefs about mental health [6, 7]. It would be important to understand HCP’s views in relation to barriers that may exist for ethnic minority women.

To date, there has been no qualitative research conducted specifically looking at different healthcare professionals’ views of how Black and South Asian women experience the access to, and acceptability of perinatal mental health care services. 

## Methods

### Aim

This study aims to develop an understanding of healthcare professionals’ views on the barriers to and facilitators of Black and South Asian women accessing and accepting perinatal mental health services.

### Design

This research has been developed as part of a wider National Institute Health Research (NIHR) project entitled: “Accessibility and acceptability of perinatal mental health services for women from Ethnic Minority groups (PAAM)” (grant 17/105/14: 2019—2023). The larger project in which this study was situated aimed to explore the views of women who engaged with PMHS, women who did not receive support from PMHS and family members who supported their loved one in PMHS. The current study focused on healthcare professionals’ views only. In this qualitative study, data were collected using one-on-one, semi-structured interviews. The protocol was pre-registered on the Open Science Framework: https://osf.io/s94bp/.

### Sampling

Purposive sampling of HCPs was used to obtain a cohort from a range of professional backgrounds including psychiatrists, psychiatric nurses, GPs, health visitors, obstetricians, social workers and midwives. The sample was representative as the HCPs interviewed were those who worked with women along the healthcare pathway from early pregnancy. This provided rich information to understand where barriers may exist at each time point. The inclusion criteria for the study required participants to meet three requirements.

- 1. Participant is willing and has the capacity to give informed consent for participation in the study.
- 2. Have a minimum of 6 months’ experience of working with women who experience mental health problems in the perinatal period.
- 3. Aged 18 or above.

### Recruitment

Recruitment for the interviews took place between January 2020 and September 2021. Healthcare professionals were approached via study collaborators and researchers from specialised perinatal mental health teams and asked if they wished to participate in the study. Professionals were contacted via email and phone and were offered the opportunity to take part in the study. The study was also advertised to healthcare professionals via social media including Instagram and Twitter. Those interested in taking part were provided with an information sheet and offered an interview slot at a date, time and location that was convenient for them. 

Ethical approval was obtained from the Health Research Authority via Queen Square Research Ethics Committee (REC reference 19/LO/1830) and governance approval was obtained from Research and Development departments in associated trusts. Written informed consent was obtained from all participants.

### Materials

The topic guide for the semi-structured interview explored HCPs’ experience of caring for women with perinatal mental illness, in particular women from ethnic minority backgrounds. HCPs were asked about patients’ referral pathways, the barriers to and facilitators of access to care for South Asian and Black women and ways to improve engagement with perinatal mental health services. This topic guide was developed with members of the research team and an advisory team made up of people with a lived experience of perinatal mental illness and those with clinical experience. The team was ethnically diverse. The topic guide used is available in Supplementary Material 1.

### Data collection

Interviews were held either online or face-to-face dependent on the participant’s preference and COVID-19 regulations at the time by four members of the research time (KB, SB, HKS, KP). Prior to the interview, informed consent was obtained and a demographic form was completed.

### Data analysis

Interviews were audio-recorded and transcribed verbatim by an external NHS-approved transcription agency. Data saturation was discussed in regular meetings with the research and supervisory team, and as the frequency of novel ideas rapidly decreased, the team considered thematic saturation to have been reached [8]. Qualitative data were analysed using Ritchie and Spencer’s [9] framework method analysis, a flexible and systematic matrix-based method with five steps. Three members of the research team (KB, SB and JFR) familiarised themselves with a selection of transcripts by reading and re-reading them (familiarisation). All were trained in qualitative data analysis, coding and the framework method. An initial framework was developed based upon emerging themes, and the existing topic guide (identifying a framework). The framework was systematically applied to the transcripts; naturally, the framework adapted as new codes emerged from the data. An example of the adapted framework is available in Supplementary Material 2. Examples of indexed data are available in Supplementary Material 3. Data were summarised in a matrix to view the emerging data (charting). KB and SB met regularly to review the charted matrix and develop emerging themes (mapping and interpretation) and discussed theme titles with MC and JJ. These themes were regularly reviewed with the wider analysis team. To optimise validity, respondent validation was conducted to check the analytic interpretation with professionals in the field such as consultant psychiatrists. Regular supervision meetings were also held with experienced qualitative researchers (RM and AC).

The rationale for using framework analysis was so that the data obtained could be analysed with a view of informing pragmatic change in practice. Framework analysis is particularly well suited to this form of policy-influencing research in a healthcare setting. Based on the research question, an inductive approach was taken to develop themes from the data gathered [10]. A more deductive approach was taken to help explain themes further in the analysis. This process was systematic with a clear trail to trace back to the raw data [10].

### Reflexivity

It is important to note the stance of the researcher to ensure reflexivity throughout the process [9, 10]. Reflexivity can be defined as “the analytic attention to the researcher’s role in qualitative research. A continuous self-critique and self-appraisal where the researcher explains how his or her own experience has or has not influenced the stages of the research process” [11, 12]. KB led the analysis and thus her experience is likely to have had the biggest impact on the interpretations of the findings. As a researcher who is also a woman from an ethnic minority background, there is an element of personal experience brought when conducting interviews and developing emerging themes. Dynamics may have been created during the interview where HCPs may have wanted to present their answers to the interview questions in a desirable way. KB worked within perinatal mental health services at the time of the research, which was felt to be beneficial, enabling professional and research knowledge to be coupled with experiential knowledge to complete the semi-structured interviews appropriately to truly address the research question. As a result, the data was analysed efficiently to keep the research question in sight. The reflexivity considerations of other authors who worked on the analysis can be found in Supplementary Material 4.

## Results

### Sample

Twenty-four healthcare professionals were interviewed in total via a phone call or video call. Interviews ranged in length from 38 to 55 min. Table 1 summarises the demographic characteristics of the HCP sample.

**Table 1:** Demographic characteristics of healthcare professionals

Characteristics	*N* = 24	%
Gender		
Female	22	92%
Male	2	8%
Age range		
20–29	2	8%
30–39	10	42%
40–49	11	46%
50–59	1	4%
Ethnicity		
Asian Indian	8	33%
White British	8	33%
White Irish	2	84%
White and Asian	2	8%
White and Black Caribbean	1	4%
Black African	1	4%
Asian Pakistani	1	4%
White, other	1	4%
Location		
Birmingham	14	58%
London	9	38%
Devon	1	4%
Type of healthcare professional		
Psychiatrist	5	21%
Mental health nurse	4	17%
Midwife	3	13%
Psychological professions	3	13%
GP	2	8%
Obstetrician	2	8%
Social worker	2	8%
Health visitor	1	4%
Nursery nurse	1	4%
Occupational therapist	1	4%
Healthcare service		
Perinatal mental health services	15	62.5%
Other outpatient healthcare services	9	37.5%

Three main themes were identified from the data: (1) lack of awareness and understanding of perinatal mental illness and service structure in both healthcare professionals and patients; (2) patients’ relationships with family, friends and healthcare professionals can both hinder and facilitate access to services; (3) healthcare professionals promote raising awareness, flexibility, developing shared understandings and questioning assumptions to improve the accessibility and acceptability of services. Each theme had several subthemes, as can be seen in Table 2.

**Table 2:** Themes and subthemes

Theme name	Subtheme name
Theme 1. Lack of awareness and understanding of perinatal mental illness and service structure in both healthcare professionals and patients	1.1 Cultural and spiritual attributions: “[Patients] don’t like to accept that they have depression, they don’t believe in it”
	1.2 Not knowing where to get help: “[There is] a lack of knowledge that PMHS exist in the first place”
	1.3 Remit and scope of services are unknown and misunderstood across groups: “Services could be better at explaining what is going to happen”
Theme 2: Patients’ relationships with family, friends and healthcare professionals can both hinder and facilitate access to services	2.1 Personal support networks are pivotal: Friends and family can both support or prevent patients from accessing help
	2.2 Interpreters have power to affect the patient-healthcare professional dynamic: “You do explain the confidentiality grounds but even still, it feels like an invasion”
	2.3 Peer support workers are trusted by communities: “They are a bridge to bringing people in”
Theme 3: Healthcare professionals promote raising awareness, flexibility, developing shared understandings, and questioning assumptions to improve the accessibility and acceptability of services	3.1. Work towards shared meanings between HCPs and patients: “There’s a different type of understanding of mental health and what mental health means to some cultural groups”
	3.2. Reflexive and reflective practices are needed to uncover biases: “I think people are scared to say that they have an unconscious bias”
	3.3. Services should offer choice where possible: “We’ll learn how to adapt our service for [patients’] needs.”
	3.4. Awareness campaigns about perinatal mental disorders: “It can happen to you, it can happen to me, it can happen to anyone”

### Theme 1: Lack of awareness and understanding of perinatal mental illness and service structure in both healthcare professionals and patients

#### Cultural and spiritual attributions: “[Patients] don’t like to accept that they have depression, they don’t believe in it”

HCPs may work with patients who attribute mental illness to spiritual or religious factors. This could impact the recognition of perinatal mental illness symptoms as understood in Western medicine. This may later impact the ability of patients to engage with HCPs as symptoms may be attributed to other non-medical explanations such as spirits. This has an effect along the referral process as patients may be less likely to accept a referral to PMHS.

I’ve seen quite a lot of mums from that background that live in denial. They don’t seek help. There are certain cultural beliefs. Most mums in that position, from where they come from, and like where I come from, they don’t like to accept that they have that depression. They don’t believe in it.—HCP017 (Health Visitor)

#### Not knowing where to get help: “[There is] a lack of knowledge that PMHS exist in the first place”

A perinatal psychiatrist suggested there is “a lack of actual knowledge that Perinatal Mental Health Service exist in the first place” (HCP010). Other HCPs also discuss the lack of knowledge HCPs may have about the symptoms of a perinatal mental illness. These may be dismissed as baby blues or other feelings patients expect to be common and pass with time. This lack of understanding can impact the ability of patients to recognise symptoms to self-refer or seek support through their GP, who tends to be their first point of contact with healthcare services. Therefore, the lack of awareness of sources of support constitutes a barrier to accessing services for patients.

Just even knowing that the services exist, or you know, the way in which mental health is spoken about within the home, or is recognised in itself, probably places a barrier, in terms of those women accessing support, knowing when they need to access support, and knowing when things are becoming, you know, unsafe, or becoming a problem—HCP005 (Nurse)

HCPs of different professions working in and out of perinatal settings all echoed the sentiment that the lack of awareness of services’ existence acted as a barrier for many of their patients.

#### Remit and scope of services are unknown and misunderstood across groups: “Services could be better at explaining what is going to happen”

There is a lack of knowledge of what perinatal mental health services offer once patients are referred or are being seen by services and what continuity of care looks like. HCPs described a common misunderstanding of the focus of perinatal services. They said that many patients feared services were there to take their child away, and said many patients felt unable to disclose struggling in case it led to the child being removed. Another important factor described was patients being referred who do not know about the referral. All these factors impact the acceptance of a referral and active engagement with services. HCPs from perinatal mental health services recognise that other services and HCPs may not know about perinatal mental health services which can impact the ability for patients to receive timely support for their mental health problem. HCPs said they often were not sure who to signpost to and therefore referrals can be back and forth between professions, leaving patients without support.

I guess services could be better about explaining exactly what’s going to happen. I know … you have a visual what happens during the ward, I think it probably would be really helpful to know exactly what's going to happen when you go and meet somebody for the first time for the initial assessment and what questions you’ll be asked. I think that might be useful.—HCP002 (Psychologist)

The lack of understanding went in both directions, with HCPs suggesting they did not know how to communicate the questions on their forms to their patients in ways that were mutually understandable. Some HCPs discussed the use of assessments and how they may not be clear or understood universally. This could be in relation to the language used to describe mental health which may not have been explained, or translated appropriately, and thus were not understood. The Western medical, highly clinical language was identified as exacerbating this lack of understanding.

I feel like the outcome measures are really clinical. So they have quite complex words for it. Some people don’t even understand, I know when I’ve done outcome measures and people always ask, what does this mean? Or I don’t know the answer of this? And sometimes it’s just you can’t really put zero on there because it effects the scores. So you’re thinking as a professional yourself, oh God what box do I tick for them.—HCP006 (Nursery Nurse)

### Theme 2: Patients’ relationships with family, friends and healthcare professionals can both hinder and facilitate access to services

#### Personal support networks are pivotal: friends and family can help and prevent people from accessing help

Family and friends can be important facilitators or barriers to patients accepting and accessing services. They are important facilitators as they can support HCPs to monitor the patient to pick up signs of deterioration and to support patients to engage with mental health services.

When she [the patient] experiences deterioration in her mental state, dad is the one that will, we can rely on, that we can contact and say, dad, we’re really worried about our patient, your daughter, can you help us?—HCP009 (Social Worker)

On the other hand, family and friends could be gatekeepers to patients initiating contact with services. There may be a fear of their community knowing about their mental health problem and a fear of what their wider family will think about their mental health problem. Family members may minimise or not discuss mental health problems which feed into stigma and therefore are a barrier for patients to engage in services.

Family can prevent people from accessing help. I think that is something that’s come up in sessions, an idea that it’s helpful to keep things within family and that you sort things out within your family and that’s it.—HCP002 (Psychologist)

…two out of the eight ladies that I spoke to had difficulties in accessing mental health, purely because their family didn’t agree with them accessing any mental health services. It’s OK if it was pregnancy, it was OK if it was medical, but mental health wise, they were kind of being discouraged away from doing that.—HCP008 (Midwife)

This led many HCPs to meet in ways that made it seem like the assessment was not about mental health. This helped patients feel safe and less anxious about having meetings. HCPs would send appointments without mental health written on the letter. It would be seen as an appointment with a midwife rather than an appointment with a mental health midwife.

I’ve had ladies on my caseloads who are Asian, not even from the Pakistani background, from like the Bengali background and I’ve been told to pretend to be a friend rather than a professional.—HCP006 (Nursery Nurse)

So we’re very careful, we don’t actually put that it’s a mental health appointment on the letters that get sent out.—HCP008 (Midwife)

#### Interpreters have power to affect the patient-healthcare professional dynamic: “You do explain the confidentiality grounds but even still, it feels like an invasion”

Several HCPs discussed different elements of communication when working with patients from Black and South Asian backgrounds who do not speak English as their first language. Language interpreters were seen as essential to facilitate discussion between clinicians and these patients. There are downfalls with both the use of an external, or a family member interpreter. An appropriate interpreter knowing the correct language and dialect was not always available. When an interpreter was available, there was a slight fear from HCPs of misinterpretation of the information shared. Furthermore, an external interpreter brings an additional person into the room which may impact patients’ ability to speak freely about their experiences and thoughts.

If somebody’s gone through something really, really traumatic it’s … the dynamics change when you’ve got an interpreter there. So really, how suitable is it to have an interpreter interpreting such potentially hugely traumatic events in that person’s life to someone? So you’ve got to think about their wellbeing [and] confidentiality.—HCP005 (Nurse)

HCPs thought language interpreters were useful to increase access to appointments. Some HCPs preferred to have an interpreter in the room rather than online or over the phone as they felt it made it more manageable as a HCP to contain.

Just using interpreters generally makes it harder, I think, to properly connect with women especially since Covid and doing things either online or over the phone.—HCP018 (Social Worker)

It’s certainly easier especially when people are very unwell to have the interpreter physically there—HCP010 (Psychiatrist)

Furthermore, HCPs mentioned concerns patients may have around confidentiality. This was for both external interpreters and family members. In relation to external interpreters, there was a fear that the interpreter would know the patient or someone in the community and information would be shared, breaking confidentiality agreements. One HCP discussed a case of this happening with a patient they worked with and the impact this had on engagement.

The person that was interpreting for her then shared information. She had worked out who the woman was, … then started sharing information about her with the Gujrati community in [CITY NAME]. So then that completely lost her faith then in ever using interpreting services again. Then we had to try and get by without one. , Using interpreters can cause a lot of challenges—HCP018 (Social Worker)

HCPs discussed their preference to not to use a family member as an interpreter, as information may not be shared appropriately and issues around confidentiality may arise as patients may not feel comfortable to disclose thoughts and feelings in front of family members, especially about those family members.

… I remember a few times with this one patient and her auntie was the interpreter so she would always be allocated to her. But her auntie wouldn’t really say to her team that I know this lady. (…) The patient would say, I don’t want the interpreter for today and it [was] too short of a notice to book another one. So then we have to rebook the appointment again and it’s just a nightmare. So I feel like the interpreting service is really bad.—HCP006 (Nursery Nurse)

#### Peer support workers are trusted by communities: “They are a bridge to bringing people in”

Peer support workers help bring patients who are in distress into services. They may come from third-sector organisations and act as a bridge into perinatal mental health services and can also support patients in third-sector organisations if they do not meet the threshold for NHS specialist perinatal mental health services. Peer support workers can also be a bridge as an interpreter (i.e. bilingual support workers exist in some services) but with an additional layer as they are someone from a similar ethnic background who has gone through a similar mental health experience and which can away the need for an external or family interpreter. Consideration does need to be given to the wellbeing of the peer support workers as they are in a potentially vulnerable position when talking to their own communities about their own experiences.

In addition, patients themselves can help to develop services using their experiences by working with staff. HCPs suggest it is important to listen to patients’ stories firsthand to improve and develop services, and their involvement can make generic training more effective.

Having peer support workers is a recovery-based initiative and it is fantastic because it really does bring hope to people.—HCP001 (Nurse)

… trying to involve the peer support workers really at every stage to ensure, try and ensure engagement, make the woman feel relaxed. …. This is our everyday bread and butter work but it’s not for other people. So I think we, kind of, need to, to, sort of, acknowledge that with, with people and, and put measures in place to put them at ease.—HCP009 (Social Worker)

### Theme 3: Healthcare professionals promote raising awareness, flexibility, developing shared understandings and questioning assumptions to improve the accessibility and acceptability of services

#### Work towards shared meanings between HCPs and patients: “There’s a different type of understanding of mental health and what mental health means to some cultural groups”

There needs to be a shared meaning and understanding between HCPs and the patients they work with in relation to their mental health, culture and ethnicity. HCPs suggest there is not “a one size fits all” approach and warned against generalising or assuming anything about any individual patient because of their culture or ethnicity. HCPs recognise that women need to be considered on an individual and holistic basis. HCPs also felt it was important that assumptions were not made about women’s childrearing practices, or how they would see the role of being a mother. This should all be considered on an individual basis and differences between western and non-western models should be respected. HCPs reflected on their own experiences and said they were careful not to impose their own cultural standards on their patients. HCPs are also reflective about their own background and experiences and the impact this can have on the dynamic with their patients.

There’s obviously a lot of heterogeneity between the different, you know cultures, we can’t quite you know, we can’t really lump it altogether but there is also a lot of common themes.—HCP007 (Psychiatrist)

HCPs emphasised how patients might find it difficult to express their feelings in the language that HCPs are used to. They emphasised how important it is to meet a patient where they are, to reflect their language back to them and to work towards a shared understanding without making assumptions that patients understand their terms for mental distress, or that they are understanding their patients’ use of language to describe distress.

… when I work with women who come from minority ethnic communities, I will have a, we will find a way of talking about similar things but in very different terms.—HCP002 (Psychologist)

#### Reflexive and reflective practices are needed to uncover biases: “I think people are scared to say that they have an unconscious bias”

HCPs recognised that they have pre-existing biases that can impact the care given to patients during the perinatal period. Examples are within multidisciplinary team decisions, interactions with patients and within the mental health system. Assumptions can be made by HCPs which can impact patient’s care, for example, single parenting assumptions based on a woman’s race, or not considering the contributing factors of culture towards mental health presentation. Staff said being honest about the biases with themselves was the only way to identify them and move to removing them.

So I think it would be naïve to suggest that were not, that we don’t, that some of these biases are, aren't playing out at times, however much we try and be on top of them. And it’s not something we really talk about either—HCP001 (Nurse)

Due to stress, HCPs working in services may become less aware of their own practices and how their unconscious biases may play out—which may normally have been picked up on. Biases also exist if services are not tailored to meet the needs of patients.

I think having support as a team to work with it, I think would be very helpful, having supervision to think about our own unconscious biases.—HCP002 (Clinical Psychologist)

Having protected time and dedicated training focused on these topics was also recommended by HCPs as a way to ensure the best care was provided to all patients.

I feel like there needs to be a lot of training in our team to understand each other’s cultures and different cultures that we may not have heard of. So I feel like if we had this proper training I feel like people’s views would definitely change. So maybe getting religious leaders from different cultures to give a session to our team so we understand them further and we can ask them questions that we may not have been comfortable to ask anyone before. HCP006 (Nursery Nurse)

#### Services should offer choice where possible: “We’ll learn how to adapt our service for [patients’] needs.”

HCPs were highly reflective of the limitations of services in supporting the communities that they are serving and argued that the onus must be on services to adapt to be able to provide the best support possible to all patients. They were wary to not put the blame on patients for not accessing services and took responsibility for the ways services fell short. HCPs felt patients’ preferences should be considered when developing services. Some HCPs thought that patients from ethnic minority backgrounds may prefer to have a HCP from the same ethnic minority background. It is also suggested that HCPs who are from the same background may understand the patient’s culture better in relation to their mental health. Patients may also assume that the staff member will not understand and might therefore talk about their experiences or culture in a superficial way.

The experiences that patients have generally had is that they will see someone that’s not from their culture who wouldn’t understand, so that is another reason that people don’t access therapies, because they will see someone who doesn’t understand why their mother-in-law is so involved in baby’s care—HCP004 (Psychological Profession)

On the other hand, two HCPs discussed their experiences of working with patients who may prefer to work with a HCP from a different ethnic/cultural background due to the fear of being judged. HCPs describe the views of some of their patients who wanted clinicians from a different cultural background:

I want someone who’s not from my cultural background because I want them to see how terrible my situation is and I want them to support me with that whereas the person, the therapist from my cultural background will judge me just like the rest of my culture judges me.—HCP004 (Psychological Profession)

Where possible, HCPs felt patients should be provided with a choice and given support to engage family members in services. It was also suggested that training in culture and religion should be provided to staff. HCP's are aware that trust can be increased through positive experiences, and thus gradually patients feel more comfortable sharing their experiences and beliefs.

#### Awareness campaigns about perinatal mental illness: “It can happen to you, it can happen to me, it can happen to anyone”

HCPs suggest that awareness should be raised about perinatal mental illness and services that exist to support these patients and their families. Suggestions given were in relation to public health campaigns on the media such as television, social media and in local communities. Some HCPs discussed their experiences of engaging with local communities such as third sector organisations or religious communities. HCPs acknowledged barriers that exist during this process, such as the dismissal of mental health problems in the local community and the resource of staff and time required to build the relationship. It was also noted by HCPs that resources should be translated into common local languages the communities speak and be accessible for patients who may not be able to read the language.

… maybe we do need more culturally sensitive information or awareness to be aimed specifically for communities. And it’s whether those communities would be accepting of a big public sort of awareness campaign, or actually it’s better aimed in more local, than national… I don’t know whether… targeting this in a more local way is more appropriate, acceptable.—HCP008 (Midwife)

## Discussion

The aim of the study was to develop an understanding of healthcare professionals’ views on current accessibility and acceptability of perinatal mental health services, and ways of improving services’ accessibility and acceptability for Black and South Asian women. Three key themes were identified. HCPs described a lack of awareness and understanding of perinatal mental illness and service structure in both healthcare professionals and patients. They identified patients’ relationships with family, friends and healthcare professionals as key: both a potential hinderance and facilitator of access to services. HCPs promoted raising awareness, flexibility, developing shared understandings and their own questioning assumptions as essential to improve the accessibility and acceptability of services to Black and South Asian Women.

The study results highlighted key barriers and facilitators for patients from South Asian and Black ethnic minority groups to access and accept perinatal mental health services. Overall, a key message was that “a one size fits all” approach will not work. HCPs were conscious not to stereotype or assume patients who share an ethnicity share ways of living their lives, or raising children. Assuming anything about a patient was identified as a potential barrier. A holistic approach was recommended, meaning that HCPs felt they should consider different elements of someone’s culture, ethnicity or spirituality in relation to their mental health treatment on an individual basis. This is also evident in the literature, as a pilot study in the USA suggested a holistic approach including peer support workers improved accessibility of services [13].

These findings should be considered in the macro context of the UK’s rapid expansion of perinatal mental health services. Increased investment in recent years means that structures of services have changed and may have been in flux at the time of the interviews. It is understandable that patients are not aware of the new service organisation in this context, as even their HCP who are in services are not confident they understand or know how to navigate referral pathways.

On an even wider level, more research in perinatal mental healthcare should make efforts to include patients from ethnic minority backgrounds, as mental health research more broadly tends to underrepresent people from ethnic minority backgrounds. In the UK, this may, in part, be attributable to the lack of legislation which mandates the inclusion of ethnic minority people in research [14]. A recommendation was made to represent these populations by providing a sample focus in research [14].

Focusing on ethnic minority patient needs in perinatal services, research has suggested that healthcare professionals such as General Practitioners (GPs) should require patients to have access to culturally sensitive, community-based perinatal mental health services, translation services and evidence-based psychological interventions in the perinatal period [15]. Templeton et al. [16] identified factors which impacted how ethnic minority patients with postnatal depression accessed healthcare. These included a lack of information on postnatal depression and services organisation, language barriers and somatisation of symptoms [16]; these broadly fit with the findings of this paper. Despite these findings, ethnic inequities in access to support persist. Research with a focus on implementation is needed to investigate why the knowledge acquired in research is not being fed back into practice and creating change.

These findings should be considered alongside, and as complementary to, research exploring Black and South Asian women’s experiences of support from perinatal mental health services. This study found that women who did access services encountered complex interlayer barriers, and many struggled to access support from services despite repeated insistent attempts [17].

HCPs mentioned adaptations they made to their practice to ensure patients were comfortable, such as not mentioning that appointments were for their mental health whenever possible. Further, HCPs said that translated information should be provided. Research has investigated the validity of perinatal assessments in a range of languages [18, 19]. There would be a question of prioritisation of the languages that would be made accessible in assessments and to also ensure sufficient understanding of specific cultural contexts. Here, third-sector organisations could support services to develop assessments in languages that may be spoken locally by ethnic minority women. As indicated in the data, it is important that services meet the needs of patients to ensure they are supported appropriately.

Results showed healthcare professionals experienced patients disengaging from services and cancelling their appointment when they were given a language interpreter as there was a fear of confidentiality being broken. Healthcare professionals found patients were more accepting of peer support workers from a third-sector organisation who were from their ethnic or cultural background and also had lived experience of mental illness. It could be assumed that the same issue of confidentiality being broken would be apparent in both groups; however, this was not found through the results from the healthcare professionals’ perspective. The distinction in acceptance could be explained by social identity perspective when help-seeking. Klik et al. [20] suggest that help-seeking behaviour for a stigmatised group has a relationship with social identification. Specifically, if seeking help is seen as in conflict with a social identity, it is less likely to take place. Peer support, which has a long history of being practiced in mental healthcare in informal ways, may facilitate a positive identity as a person that asks for help and needs support, rather than a stigmatised identity of an unwell person. Peer support promotes person-centred recovery by enabling contact between people with lived experience to foster a sense of connectedness by communicating shared experiences. This may strengthen social identification. Patients who identify positively with the peer support workers may be more accepting of a referral as they belong to that social identity of being from the same cultural or ethnic background with a mental illness compared to being a woman working with someone who is also from the same ethnic background without the illness. Research into how the intersecting social identities of having perinatal mental illness and sharing a cultural or ethnic background interact and impact access to services is needed.

Peer support involves people who share a lived experience of mental health problems supporting others in their recovery from mental health problems, although systematic training for peer support workers is relatively new in NHS services [21], and services are not widely available. The findings point to peer support workers acting as a bridge into the services. Some HCPs mentioned that peer support workers being embedded within services across NHS trusts might support patients to maintain engagement in perinatal mental health services. This deserves further exploration, both from the perspective of services, patients and peer support workers as there is little information on this in perinatal settings. A recent systematic review of implementation of peer support work identified no studies conducted in perinatal settings [22]. One study conducted by Biggs and colleagues (2018) looked at peer support workers’ experiences supporting an Australian perinatal mental health helpline [23]. It was found that these peer supporters felt personal benefits such as feeling motivated to help others and make a difference. It would be interesting to see the experience of peer support workers in the NHS in perinatal mental health services especially as they may work alongside staff who may have once cared for them. This may be an avenue to ensure the persisting health inequalities observed between women who belong to ethnic minority groups and White British women could be eradicated.

The results indicated there is a lack of awareness of perinatal mental illness and this awareness needs to be raised through local or national interventions. Knifton et al. (2009) suggest community approaches to tackle stigma are more valuable than top-down public education. This supports our results and indicates that local interventions may be more appropriate compared to large public health interventions [24]. HCPs discussed how important it would be to inform other HCPs about the referral pathway for perinatal mental health services. This would help support patients into services which may often be fragmented when there is no clear route. Awareness needs to be increased within services, as well as within the general public and specific communities who are currently being left suffering in silence.

The findings of this research have several implications for service organisation and clinical practice. HCP identified barriers and solutions that ranged widely, including having interpreters more readily available, improving training for interpreters and using peer support workers. Each of the suggestions that arose from this qualitative work should be explored in other study designs. In addition, the findings of this study should be considered in parallel and compared to solutions suggested by patients from ethnic minority backgrounds: the comparison, and possible discrepancies would be interesting to delve into. As we interviewed a range of HCPs, we could identify key areas that were working well in perinatal mental health services to support engagement for patients and see areas that may not have been consistent between professions.

### Strengths and limitations

This study has several strengths. To the authors’ knowledge, it is the first qualitative study to explore the views of multiple healthcare professionals about access to and acceptability of care for ethnic minority women. It addresses an important gap in mental health service delivery from a unique perspective: the barriers to access for ethnic minority women in a large sample of diverse professionals.

Another important strength is the study team which included a wide range of professionals including perinatal clinicians, people with lived experience of perinatal mental illness and with diverse ethnic backgrounds. The study was conducted with consistent input from a Lived Experience Advisory Panel from design through to interpretation of the results.

A limitation of the current approach is that peer support workers were not interviewed. Their perspective would have been a great addition; however, when the study was conducted, the roll-out and integration of peer support workers in perinatal services was in its infancy. Although peer support workers were not excluded, we did not have any come forward as eligible. As peer support workers work at the intersection of health services and community organisations and because of the newness of the role, they could not be included in the current analysis. Future research should explore their perspectives on how to redress inequalities in access to perinatal mental health services.

Another limitation was the topic guide which, on reflection, may have included some leading questions. For example, questions around bias were asked in the following manner “Do you think you/other healthcare professionals could have an unconscious bias that could act as a barrier for ethnic minority women?” This could have led to a desirability bias effect where HCPs portrayed themselves as behaving in the way they would have liked to behave. This therefore limits the generalisability of this particular result. More research in which HCPs were observed, or more time was given to develop trust with the interviewer, might have provided different findings.

Another limitation of this study was that the sample was not large enough to be able to determine differences between HCP professions or services. Therefore, we were unable to analyse the data on a level which may have provided further insight into discrepancies between different HCPs or services.

Finally, only two of the sample interviewed were men. More women than men work in perinatal settings and in mental healthcare more broadly; however, the split is not as biased towards women as the sample in the current study [25]. It is possible the views held by men professionals differ to women’s, and these are under-represented in this sample.

## Conclusion

Healthcare professionals’ views on how to support South Asian and Black women to access perinatal services and improve their experience of care in services are summarised in this paper. The findings confirm the existence of barriers for South Asian and Black women, specifying these as related to factors in awareness of PMI and PMHS from HCPs to patients, services being appropriate for the communities they serve and the impact of HCPs and support systems to support women into PMHS. Future research can employ observational ethnography so that staff interviews can be triangulated with observation of staff working with women from ethnic minorities; this will allow us to better understand the link between staff views and attitudes and actual experiences. In addition, research could look at peer support workers across perinatal mental health services. It would be beneficial to understand their experiences of working in perinatal mental health services and gaps for development. Future research could also include views from other HCPs in other healthcare services such as accident and emergency departments and maternity departments. It would be important to understand the views of staff members who may not have speciality training but see women along the pathway of their perinatal journey to have a clinical picture. As mentioned in the design, this research has been developed as part of a wider NIHR funded project. Data has been collected from women who disengaged from services, women who engaged in services and their family members to further understand their views of perinatal mental health services. These results in conjunction with the findings from HCPs could contribute towards developing existing services and shaping future research.

### Supplementary Information

**Additional file 1.**

## Data Availability

The datasets generated and analysed during the current study are not publicly available to maintain participant anonymity but are available from the corresponding author on reasonable request.

## Abbreviations

HCP: Healthcare professional
PMH: Perinatal mental health
PMHS: Perinatal mental health services

## Glossary

Peer support worker: Peer support workers are people who have lived experience of mental health challenges themselves, working within services and using these experiences, and empathy, to support other people and their families receiving mental health services [26]
Culture: The distinctive customs, values, beliefs, knowledge, art and language of a society or a community. These values and concepts are passed on from generation to generation, and they are the basis for everyday behaviours and practices [27]
Ethnicity: Belonging to a group identity based shared on culture, religion, traditions and customs [28]
