# Supplementary material for: Healthcare professionals’ views on the accessibility and acceptability of perinatal mental health services for South Asian and Black women: a qualitative study
Source: BMC Med. 2023 Oct 2;21:370. doi: 10.1186/s12916-023-02978-5 (PMC10546637; doi:10.1186/s12916-023-02978-5)
Supplement: Supplementary file 1 — Additional file 1. [file 12916_2023_2978_MOESM1_ESM.docx]

## Supplementary Material 1. Topic Guide

**Topic guide: Qualitative interviews for PAAM (WP3):**

**Healthcare professionals**

1. **Introduction**

- Introduce self
- Explain nature and purpose of the research
- Provide assurances about confidentiality and no link to current treatment/care (e.g. your name and information will only be known by the researchers and will not be revealed to anyone else)
- Explain what happens to data collected – recording, transcribing, analysis, anonymity (all potentially identifying information will be removed), audio recordings will be destroyed once data analysis is complete, check how they would like to be referred to during recording
- Introduce tape recorder
- Explain how interview works- interviewer will not say very much and will tend to ask questions, all views important, no right or wrong answers, looking for a range of views
- To say if they are feeling stressed/uncomfortable. Do not have to answer a question or can change topic if needed. The interview can be paused at any time if you require a break.
- Mobile phones off or on silent
- Invite any questions

*Note for interviewer:*

*As far as possible make sure you probe and ask for specific examples. So, if the participant states that ‘needs specific to their culture were not met’ ask the participant to provide specific examples of this.*

1. **Background**

**🡪 *Ask participant about their professional role and about experience of working with mums with perinatal mental illness from ethnic minorities***

Potential probes

Have you experienced challenges in caring for this group of mums

*The purpose of this section is to obtain a clear understanding of the professional’s role and experience of working with mums with perinatal mental illness from ethnic minority backgrounds*

1. **Accessibility and acceptability of PMH service for mums from ethnic minorities**

**🡪 *Ask participant about their experience of a) considering referral and referring to PMH service (if non perinatal mental health specialist or***

***b) working with mums from ethnic minorities (if perinatal mental health specialist)***

Potential probes

- What is a referral pathway for a mum with perinatal mental illness in your service?
- From your experience what are the potential barriers for South Asian/Black mums with perinatal mental illness to receive care & treatment in your service?
- From your experience which factors facilitate the engagement of South Asian/Black mums with perinatal mental health teams?
- What do you think about a role of family/friends in supporting mums and helping/not helping them access PMH services?
- What factors contribute to South Asian/Black mums with perinatal mental illness disengaging from PMH service?
- From your experience how we can we make perinatal mental health services easier to access for South Asian and Black communities?
- Do you think you/other healthcare professionals could have an unconscious bias that could act as a barrier for ethnic minority women?

*By the end of this section, the interviewer should have established professionals’ experience of potential barriers and factors that facilitate receiving care and treatment for South Asian and Black mums with perinatal mental illness, and factors relevant for disengagement and suggestions for making service easier to access*

**Conclusion**

- Do you have anything else to add to what we have discussed today: any further comments?
- Thank you for your contribution

## Supplementary Material 2. Adapted Framework


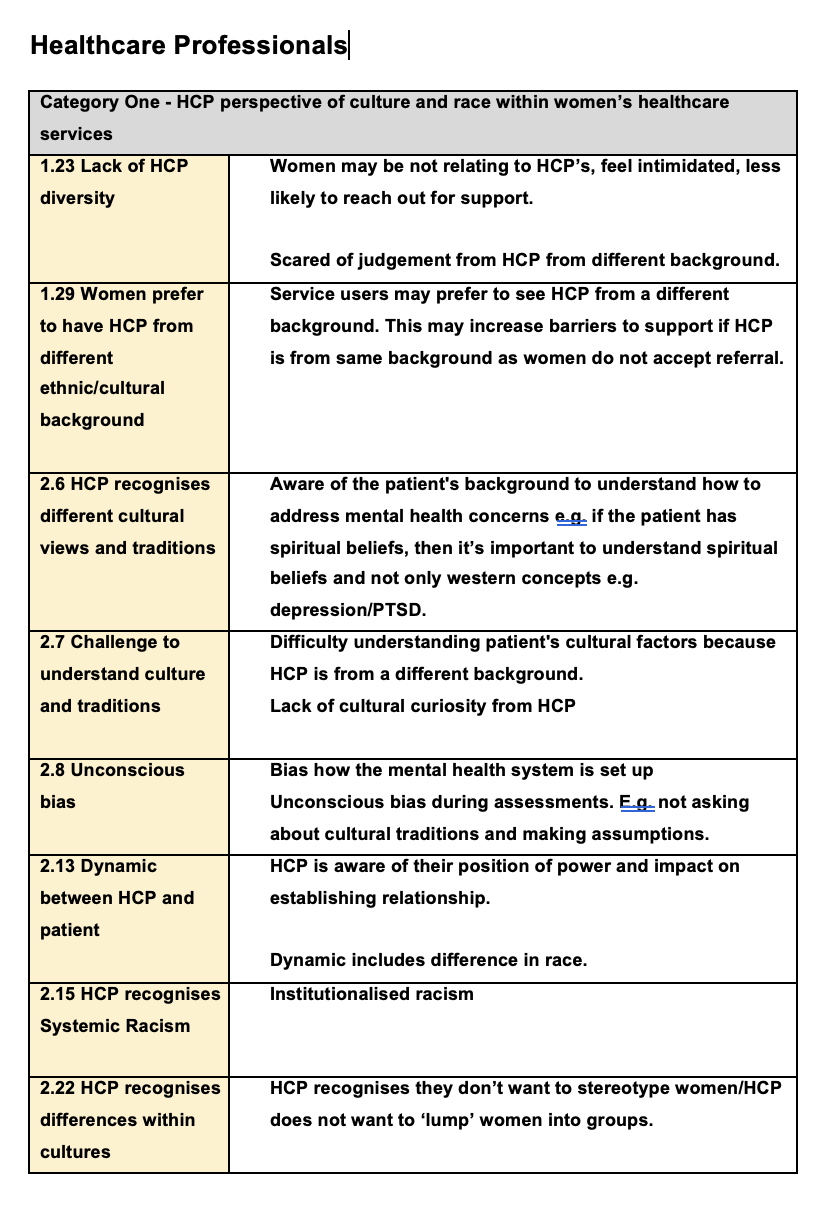


## Supplementary Material 3. Indexed Data


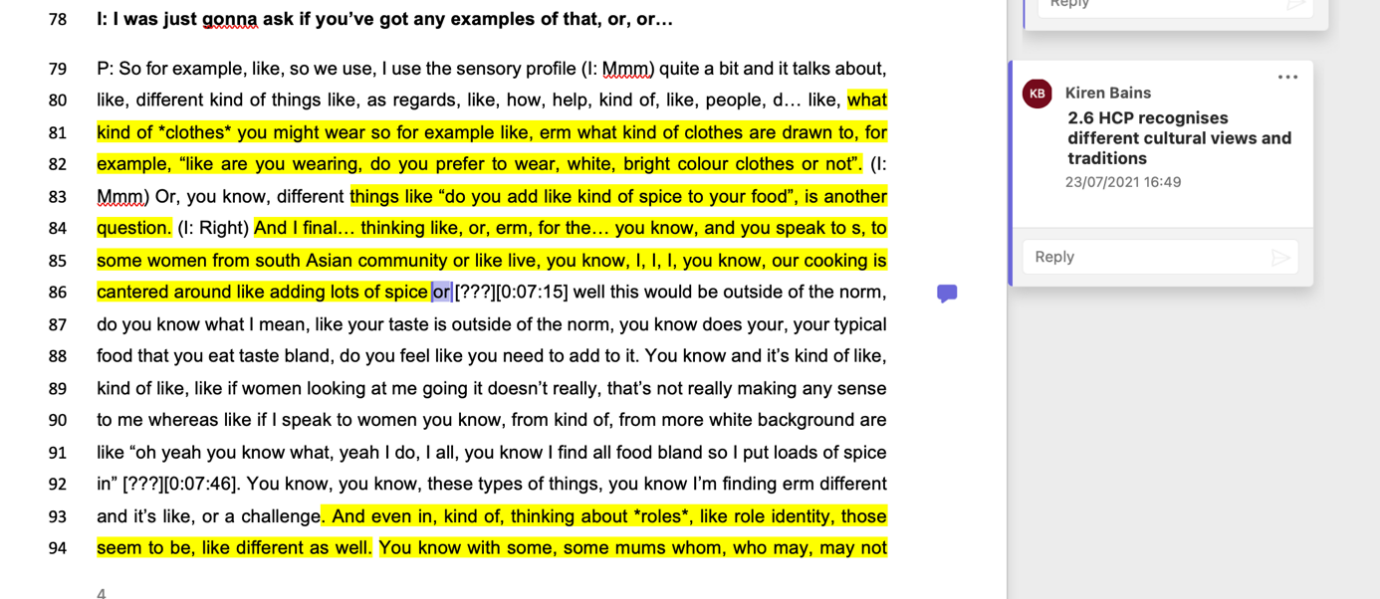


## Supplementary Material 4. Reflexivity Table

Details of researchers involved in interviewing and analysis.

| **Initials** | **Gender** | **Training** | **Credentials/Education** | **Occupation** | **Other relevant experience/views?** |
| --- | --- | --- | --- | --- | --- |
| MC | Female | Qualitative analysis trained, Interview training (Social Research Authority). Thematic analysis and Framework analysis training. | BA, MA, MSc, PhD social psychiatry. | Post-doctoral researcher | White Irish ethnicity, migrated age 18 to the UK. Interested in social belonging and its impact on health, migrants’ mental health and critical approaches in psychiatric treatment. |
| SB | Female | Post graduate training in Qualitative research including interviewing, and thematic and framework analysis. | BSc (Hons) Psychology  PGCert Applied Health Research | Research Fellow in Perinatal Mental Health | White British, born and living in the UK with no children. Background in clinical psychology and have worked in various clinical roles in adult mental health in NHS and third sector organisations, including diverse populations in Birmingham and Coventry areas of UK. |
| KCP | Female | Interviewing, analysis of transcripts and creating of matrix | BSc, MSC, DClinPsy in progress. Training from BSc and MSc in qualitative approaches | Research assistant during data collection, currently trainee clinical psychologist | White British ethnicity, born in Germany and moved to UK before 1st birthday. Interested in developmental psychology and passionate about working with children and young people and their families. |
| HKS | Female | Qualitative analysis interview training | BA, MA, PHD, AFHEA | Associate Lecturer | Academic, South Asian Britain, Indian, second generation. Interests in health inequities among ethnic minority groups in the perinatal period, lived experiences of illness and patient experiences of healthcare services. PhD in the lived experiences of South Asian women with a severe postnatal psychiatric illness and their experiences of perinatal mental health services. Previous experience of a clinical placement at a Mother and Baby Unit. |
| KB | Female | Work as a clinical studies officer.  Training from BSc and MRes in qualitative research methods.  Training and delivery of focus groups, delivering interventions and data collection from one-to-one interviews with children. Training and experience of structured and semi-structured interviews. | BSc, MRes in progress | Clinical Research Practitioner | Researcher born in the UK but from a South Asian background. Previous work experience was in Sri Lanka in a mother and baby unit which sparked interest of perinatal services in the UK and wanting to learn more about the services offered in the UK. Worked as an honorary assistant psychologist in perinatal mental health services which increased awareness of the running of the service locally. |
